# Chemical Post-Processing of SLM Ti6Al4V Structures Using HF with Propylene Glycol: Surface, Structural and Biological Evaluation of an Exploratory Post-Processing Strategy

**DOI:** 10.3390/ma19183924

**Published:** 2026-09-15

**Authors:** Krzysztof Jastrzębski, Klaudia Szafarz, Adam K. Puszkarz

**Affiliations:** 1 Institute of Materials Science and Engineering, Faculty of Mechanical Engineering, Lodz University of Technology, 1/15 Stefanowskiego Street, 90-537 Lodz, Poland; 2 Textile Institute, Faculty of Textiles and Design, Lodz University of Technology, 116 Żeromskiego Street, 90-924 Lodz, Poland; adam.puszkarz@p.lodz.pl

**Keywords:** post-processing, additive manufacturing, Ti6Al4V, selective laser melting, chemical etching, biocompatibility, micro-CT

## Abstract

**Highlights:**

- Novel HF–propylene glycol etching approach for post-processing of SLM-fabricated Ti6Al4V.
- Propylene glycol reduced HF-induced mass loss of SLM Ti6Al4V by up to 50%.
- HF–propylene glycol solutions limited fluorine incorporation into the surface.
- Modified surfaces showed no cytotoxic effects toward osteoblast-like cells.

**Abstract:**

Selective Laser Melting (SLM) of Ti6Al4V produces implants with surface-adhered, weakly bonded powder particles that require post processing prior to biomedical use. Chemical etching with hydrofluoric acid (HF) is effective but often causes excessive material loss. In this study, SLM-fabricated Ti6Al4V samples were chemically etched using 1–3% HF solutions with and without the addition of propylene glycol. The influence of etching conditions on mass loss, surface morphology, roughness, wettability, chemical composition, internal porosity (using X-ray micro- computed tomography) and biological response was evaluated. The results indicted that increasing HF concentration increased material loss and powder removal efficiency. The addition of propylene glycol substantially reduced mass loss (up to 50%) while preserving effective powder removal and surface quality. Etched samples exhibited reduced roughness parameters and no cytotoxic effects toward *Saos-2* cells. Moreover, selected etching conditions shifted the porous architecture toward pore-size distributions considered potentially more favorable for bone tissue ingrowth. These findings indicate that HF etching with propylene glycol is a promising post processing strategy for SLM Ti6Al4V implants, offering improved surface control with reduced material degradation.

## 1. Introduction

Additive manufacturing (AM) enables fabrication of patient-specific implants with controlled porous architectures that promote osseointegration while maintaining adequate mechanical performance [1,2]. One of the most significant advantages of additive manufacturing lies in its ability to precisely control the size, morphology, and spatial distribution within fabricated structures [3,4]. This capability is critical for biomedical implants, as porosity plays a central role in promoting tissue integration by facilitating angiogenesis and stimulating tissue ingrowth [5]. Natural tissues, particularly bone, exhibit highly complex hierarchical architectures characterized by multi-scale porosity [6,7]. Reproducing such intricate structural features remains a major limitation of conventional manufacturing routes. Beyond geometric design, the mechanical properties and fatigue behavior of additively manufactured components are crucial factors determining their suitability for biomedical applications. These properties depend, among other factors, on post-processing treatments applied after fabrication. Surface modification methods are widely employed to adjust surface roughness, porosity level, and surface energy, all of which significantly influence biological responses at the material–tissue interface [8].

Despite these advantages, SLM-fabricated Ti6Al4V structures exhibit surface-adhered partially melted powder particles, irregular surface morphology and geometrical deviations that require post-processing before biomedical application. Powder-based additive manufacturing technologies, such as selective laser melting (SLM) or electron beam melting (EBM), inherently result in residual powders remaining on or within the surface of fabricated components. These particles may remain loosely attached to the surface or become entrapped within internal pores, posing a risk of uncontrolled particle release into the surrounding environment—an especially critical issue in medical and biomedical applications. Consequently, post-processing procedures aimed at removing residual powders and modifying surface characteristics are essential. Currently, post-processing of metal AM parts typically involves mechanical techniques (e.g., vibration or blasting), electrochemical polishing, and thermal or pressure-assisted treatments [9,10,11,12], however, chemical etching remains particularly attractive for porous structures because it can penetrate complex internal geometries while simultaneously removing loosely bound powder particles.

Hydrofluoric acid (HF) solutions of varying concentrations and compositions—commonly combined with hydrochloric acid (HCl) or nitric acid (HNO_3_)—are widely reported as effective agents for post-processing of additively manufactured titanium components [13,14,15,16]. HF-based solutions are also commonly used in electrochemical surface modification of titanium, particularly for the formation of titanium oxide nanotubes. In such processes, electrolyte systems containing HF and organic solvents, such as glycols, are frequently employed [17,18]. To the best of our knowledge, despite their widespread use in electrochemical anodization, HF–glycol-based solutions have not been systematically investigated as chemical etching media for post-processing of additively manufactured Ti6Al4V structures.

The aim of this study was therefore to evaluate the effect of incorporating propylene glycol into HF etching solutions on the surface properties, internal structure, and biological response of selective laser melted (SLM) Ti–6Al–4V alloy. This work introduces a previously unexplored post-processing strategy for SLM-fabricated Ti6Al4V and contributes to the development of surface modification approaches for biomedical titanium implants.

## 2. Materials and Methods

### 2.1. Material and Sample Fabrication

Samples made of Ti6Al4V ELI were manufactured using Selective Laser Melting (SLM) technology. The specimen geometry was provided by Medgal Ltd. (Księżyno, Poland) and corresponds to the design used for personalized orthopedic implants. The powder used was LaserForm Ti Gr 23. Printing was performed on a ProX 320A (3D Systems, Rock Hill, SC, USA) system equipped with a build platform of 275 × 275 mm. The manufacturing parameters are summarized in Table 1. Commercial powder—LaserForm Ti Gr 23. Type A (3D Systems GmbH, Germany) was used as the feedstock material. Additional processing parameters, including scanning speed, hatch spacing, energy density etc. constitute proprietary know-how of the manufacturer and were therefore not available for disclosure.

**Figure 1:**
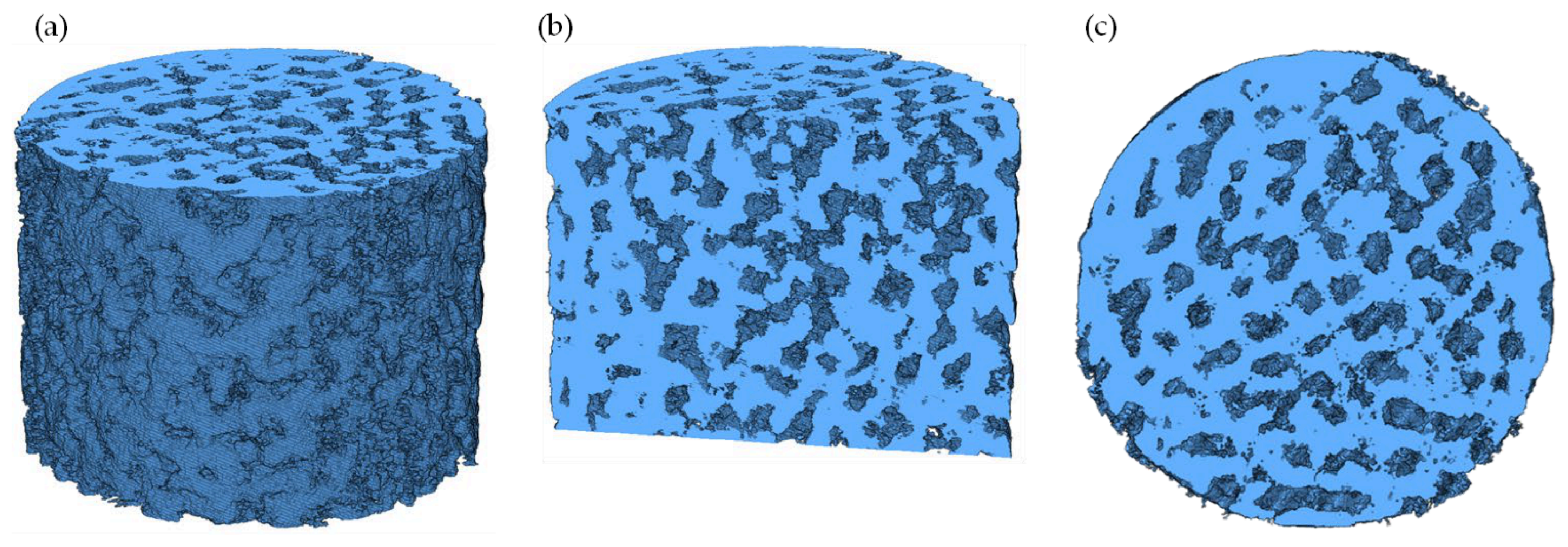
3D micro-CT visualizations of untreated sample: (**a**) general view, (**b**) vertical cross-section, (**c**) horizontal cross-section.

Following fabrication, all samples underwent heat treatment in a high vacuum furnace at a pressure of 5 × 10^−5^ mbar, at a temperature of 920 ± 15 °C for 2 h, so as to remove the stresses related with printing process. After heat treatment, the samples were removed from the build platform. Two types of specimens were prepared: samples with a highly developed porous surface representative of orthopedic implant structures—used for biological and structural assessment and solid samples used for surface roughness and wettability measurements. All samples had a cylindrical geometry: diameter equal to 10 mm and a height equal to 7 mm. The visualization of unmodified sample is presented in Figure 1.

### 2.2. Mass Loss Measurement

To determine the material loss resulting from chemical etching, samples were weighed before and after the chemical treatment. For this purpose 415 MA110.R moisture analyzer (Radwag, Radom, Poland) was used. To eliminate the influence of residual moisture drying was conducted at 200 °C for 10 min with usingconstant drying profile. The initial and final masses of each porous specimen were determined from three consecutive weighings performed on the same specimen to verify measurement stability. Prior to initial weighing, samples were ultrasonically cleaned in isopropyl alcohol, deionized water and methanol in each case for 5 min. The final weighing was performed without repeating the initial ultrasonic cleaning sequence. Mass loss was calculated as the percentage difference between the initial and final mass.

### 2.3. Chemical Etching Procedure

Prior to chemical treatment, all samples were ultrasonically cleaned in isopropyl alcohol for 10 min and subsequently dried via compressed air. This additional ultrasonic cleaning step, immediately before etching, was introduced to remove handling-related contaminants before chemical etching. Preliminary verification performed prior to the main experiments indicated that this procedure did not produce a measurable influence on the subsequent mass-loss determination.

Chemical etching was performed using hydrofluoric acid—the stock solution (Chempur, Piekary Śląskie, Poland) was 40%, with or without propylene glycol (Centro Chem Sp. z o.o. sp.k., Lublin, Poland) as an additive. Six etching solutions were prepared: HF at concentrations of 1%, 2% and 3% (*v*/*v*), and the same HF concentrations with the addition of 1% (*v*/*v*) propylene glycol. All solutions were freshly prepared in polypropylene vessels due to the highly corrosive nature of HF toward glass.

Each sample was immersed individually in 100 mL of the etching solution and treated for a fixed duration of 300 s. Mixing was provided using magnetic stirrer working at 300 rpm in order to promote uniform solution exchange throughout the interconnected porous architecture and minimize local accumulation of reaction products within internal pores. All the processes were performed at room temperature. After etching, samples were immediately transferred to deionized water to stop the reaction. After 5 min samples were transferred to ethanol for about 30 s and subsequently dried with compressed air. The mass measurements were then conducted according to protocol presented in Section 2.2.

For each etching condition, two independent porous specimens and two solid SLM reference specimens were prepared. One porous specimen was used for mass-loss determination and subsequent Micro-CT characterization. Both porous specimens were used for SEM/EDS observations and biological evaluation, whereas the two solid specimens were used for roughness and wettability measurements.

To ensure clarity and consistency of presented data, a systematic nomenclature was adopted for all investigated samples. Samples etched in hydrofluoric acid solutions were labeled according to the nominal HF concentration used during chemical treatment (1%, 2%, or 3% *v*/*v*). Samples treated in HF solutions containing 1% (*v*/*v*) propylene glycol were denoted by the suffix “+PG”. Porous samples with a highly developed surface, representative of orthopedic implant structures, were labeled without any prefix. Solid printouts were identified by the prefix “S” (e.g., SHF 1%), indicating their non-porous geometry.

### 2.4. Surface Roughness

Surface roughness was evaluated using a Hommel Tester T1000 Wave contact profilometer (Hommel-Etamic, Villingen-Schwenningen, Germany). Measurements were carried out only on solid printouts. The roughness parameters Ra (arithmetical mean roughness) and Rz (maximum height of profile), were recorded with the help of EVOVIS Software (Hommel-Etamic, Villingen-Schwenningen, Germany)

The roughness parameters Ra and Rz were determined in accordance with ISO 4287 [19], using a Gaussian filter with a cutoff length ($\lambda_{c}$) of 0.80 mm. The evaluation length was 4.80 mm, consisting of six sampling lengths of 0.80 mm. Measurements were performed using a TK300 stylus with a 5 µm tip radius, at a measuring speed of 0.50 mm/s and a sampling interval of 0.5 µm, resulting in 9598 measurement points over a measurement range of 400 µm. Roughness profiles were acquired on the top surface of the solid SLM specimens, with the stylus traversing the surface perpendicular to the predominant surface texturing produced during the manufacturing process. Measurements were performed on two independent solid specimens for each etching condition, with ten roughness profiles acquired across both specimens.

### 2.5. Surface Morphology and Chemical Composition

High resolution observations of surface morphology were performed using a scanning electron microscope (SEM) JSM 6610LV (JEOL, Akishima, Japan) equipped with EDS X-Max 80 analyzer (Oxford Instruments, Abingdon, UK), under high-vacuum conditions at an accelerating voltage of 10–20 kV. Secondary electron images (SEI) were acquired using a spot size setting of 60. For each experimental condition, SEM observations were performed on two independently etched porous specimens, with multiple randomly selected surface regions examined on each specimen. The micrographs presented in this study are representative of these observations. Analysis covered the surface chemical composition and changes induced by chemical etching, with particular attention to the presence of fluorine and oxygen. EDS results were treated as semi-quantitative, and elemental composition was determined using the manufacturer’s standardless quantification procedure provided by Oxford Instruments software—AZtec Suite 6.0 (Oxford Instruments NanoAnalysis, Abingdon, UK).

### 2.6. Wettability and Surface Energy

Surface wettability was evaluated using the sessile drop method with a FM40 Easy Drop goniometer (Krüss, Hamburg, Germany). Static contact angle was measured 5 s after droplet deposition. Deionized water (polar liquid) and diiodomethane (non-polar liquid) were used as test liquids. A droplet volume of 0.8 µL was dispensed onto the sample surface. The contact angle was determined by fitting the droplet profile using Drop Shape Analysis software (Krüss, Hamburg, Germany), and the reported value corresponds to the average of the left and right contact angles.

For each sample (two of each kind) and each testing liquid, six measurements were performed at random locations of solid 3D printouts. The surface free energy (SFE) and its polar and dispersive components were calculated using the Owens–Wendt approach based on the measured contact angles and following Equations (1) and (2):

(1) $$\gamma_{L}\frac{1+\cos\theta }{2}={\left(\gamma_{L}^{d}\gamma_{S}^{d}\right)}^{0.5}+{\left(\gamma_{L}^{p}\gamma_{S}^{p}\right)}^{0.5}$$

(2) $$\gamma_{S}=\gamma_{S}^{d}+\gamma_{S}^{p}$$

where:

$\gamma_{S}$—surface free energy of a solid sample
$\gamma_{L}^{d},\gamma_{L}^{p}$—dispersive and polar parts of the liquid surface tension
$\gamma_{S}^{d},\gamma_{S}^{p}$—dispersive and polar parts of the solid surface energy
*θ*—contact angle at the liquid/solid interface

### 2.7. Micro CT Structural Analysis

Internal structure and porosity of the tested samples were analyzed using high-resolution SkyScan 1272 X-ray micro-CT system (Bruker, Kontich, Belgium). Scanning was conducted at X-ray source voltage equal to 90 kV; X-ray source current: 111 µA with a pixel size of 26.5 µm and exposure time of 1620 ms. An Al 0.5 mm + Cu 0.038 mm filter was applied to reduce beam hardening artifacts. Samples were rotated with a step of 0.5°, and four images were averaged per angular position. Three dimensional reconstructions were used to determine total porosity, wall thickness distribution, and inter wall distances of the porous structures using computer software: NRecon 1.7.4.2, CTAn 1.17.7.2+ and Data Viewer 1.5.6.2 (Bruker, Kontich, Belgium).

For each etching condition, one porous specimen was analyzed by Micro-CT following the mass-loss measurement. The region of interest (ROI) comprised the entire porous specimen, and the structural parameters were determined using threshold-based segmentation. The reported standard deviations represent the variability of the measured structural parameters obtained from the analyzed specimen.

### 2.8. Biological Response Evaluation via Cytotoxicity Assessment

Cytotoxicity was assessed using the XTT assay according to ISO 10993 5 [20]. Prior to testing, samples were ultrasonically cleaned in ethanol for 10 min and steam-sterilized in an autoclave at 121 °C for 15 min. Due to potential application of printouts as orthopedical implants the *Saos-2* osteoblast like cells were selected as a model cell line.

Extracts were prepared by incubating the samples in McCoy’s culture medium at a ratio of 0.1 g/mL for 48 h at 37 °C. *Saos-2* osteoblast like cells were seeded onto 96 well plates at a density of 1 × 10^4^ cells/well and incubated for 24 h. The medium was then replaced with the sample extracts, followed by incubation for an additional 24 h.

XTT reagent (Biotium, Fremont, CA, USA) was added, and absorbance was measured after 4 h at 450 nm (reference: 650 nm) using a Victor X4 microplate reader (PerkinElmer, Shelton, CT, USA). Cell viability was calculated relative to a negative control (untreated cells). As a positive control, 6% DMSO in McCoy’s medium without supplements was used. Materials were classified as non-cytotoxic when cell viability exceeded 70%.

## 3. Results

### 3.1. Mass Loss

Increasing HF concentration from 1% to 3% led to a pronounced increase in mass loss (Table 2 and Table 3). Porous samples exhibited substantially higher mass loss compared to solid specimens, reflecting their higher surface to volume ratio and increased accessibility of the etching solution. In the case of porous samples the mass loss reached up to one third and one sixth consecutively for pure hydrofluoric acid and its mixture with glycol. Reducing the HF concentration from 3% to 2% decreased the mass loss by approximately 40%, regardless of the presence of propylene glycol. For all HF concentrations, the addition of propylene glycol pronouncedly reduced mass loss. At 3% HF, incorporation of propylene glycol decreased material loss by approximately 50% compared to HF alone.

### 3.2. Surface Roughness

Surface roughness measurements performed on solid samples showed a reduction in Ra and Rz values after chemical etching relative to the untreated printouts (Table 4). Etching with HF without additives led to a gradual decrease in roughness parameters with increasing acid concentration. In contrast, samples treated with HF solutions containing propylene glycol showed a less pronounced trend, primaly due to greater data scatter. Nevertheless, surface smoothening seems regardless of the addition of propylene glycol.

### 3.3. Surface Morphology and Chemical Content

**Figure 2:**
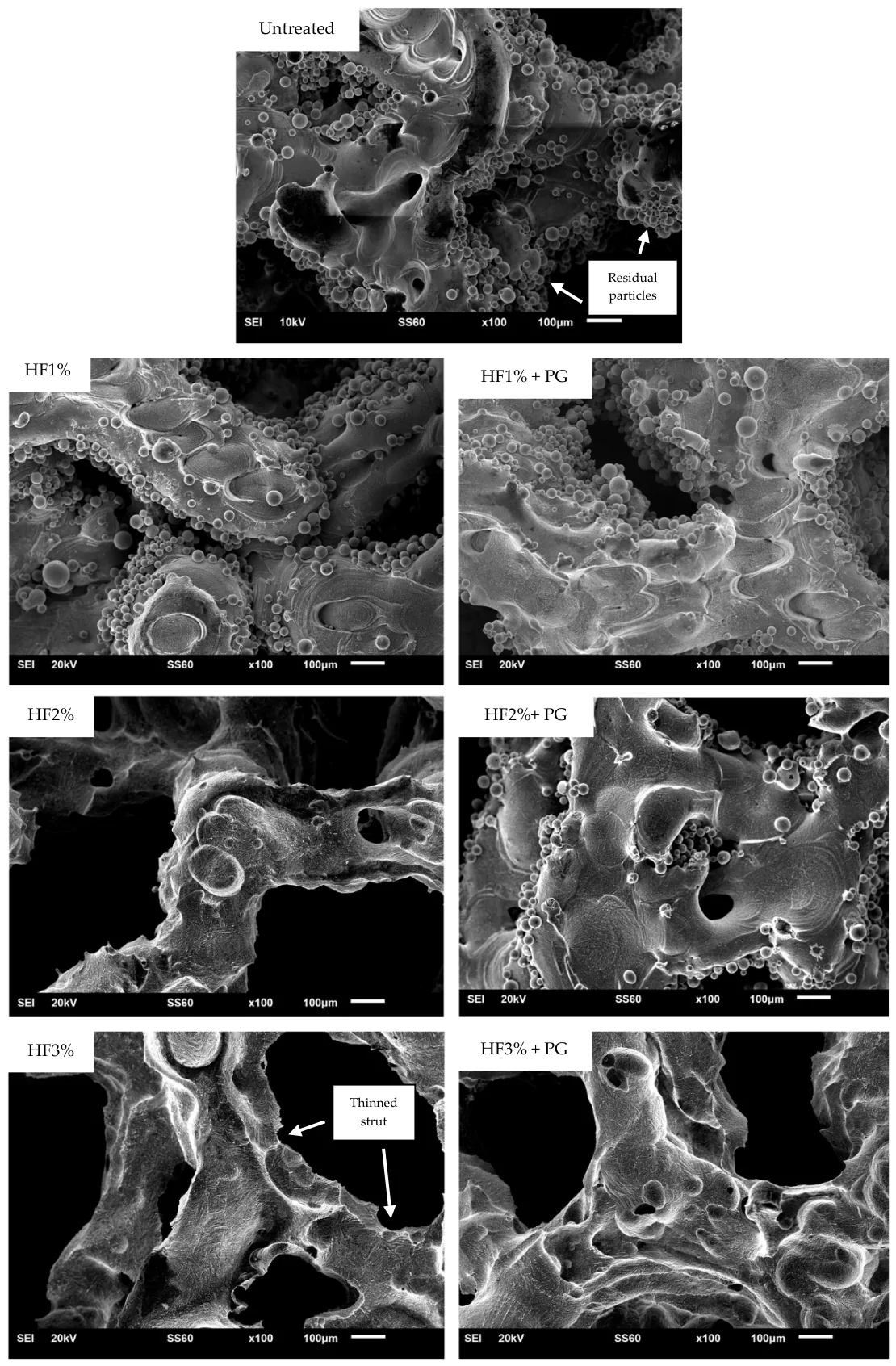
Representative SEM micrographs of the surfaces of SLM-fabricated Ti6Al4V specimens before and after chemical etching in HF solutions with and without propylene glycol, illustrating the progressive removal of loosely bound powder particles and the accompanying thinning of struts. Representative annotations indicating residual powder particles (untreated specimen) and strut thinning (3% HF) were added to facilitate interpretation.

The untreated porous samples exhibited numerous partially melted and weakly bonded powder particles, predominantly located at strut edges and junctions. Noticeable changes in surface morphology (amount of weakly bonded particles but also struts itself) induced by chemical etching were clearly visible in the case of all etched samples (Figure 2). Although the amount of attached powder was reduced for usage of 1% HF solution residual particles remain visible regardless of propylene glycol usage. The highest detected removal of weakly bonded powder particles was achieved for 2 and 3% HF solution or HF 3% + PG samples. However, porous samples etched in 3% HF without propylene glycol exhibited surface thinning and localized material loss. The addition of propylene glycol substantially reduced the occurrence of such defects, resulting in smoother and more uniform surfaces while maintaining effective powder removal for this acid concentration.

**Figure 3:**
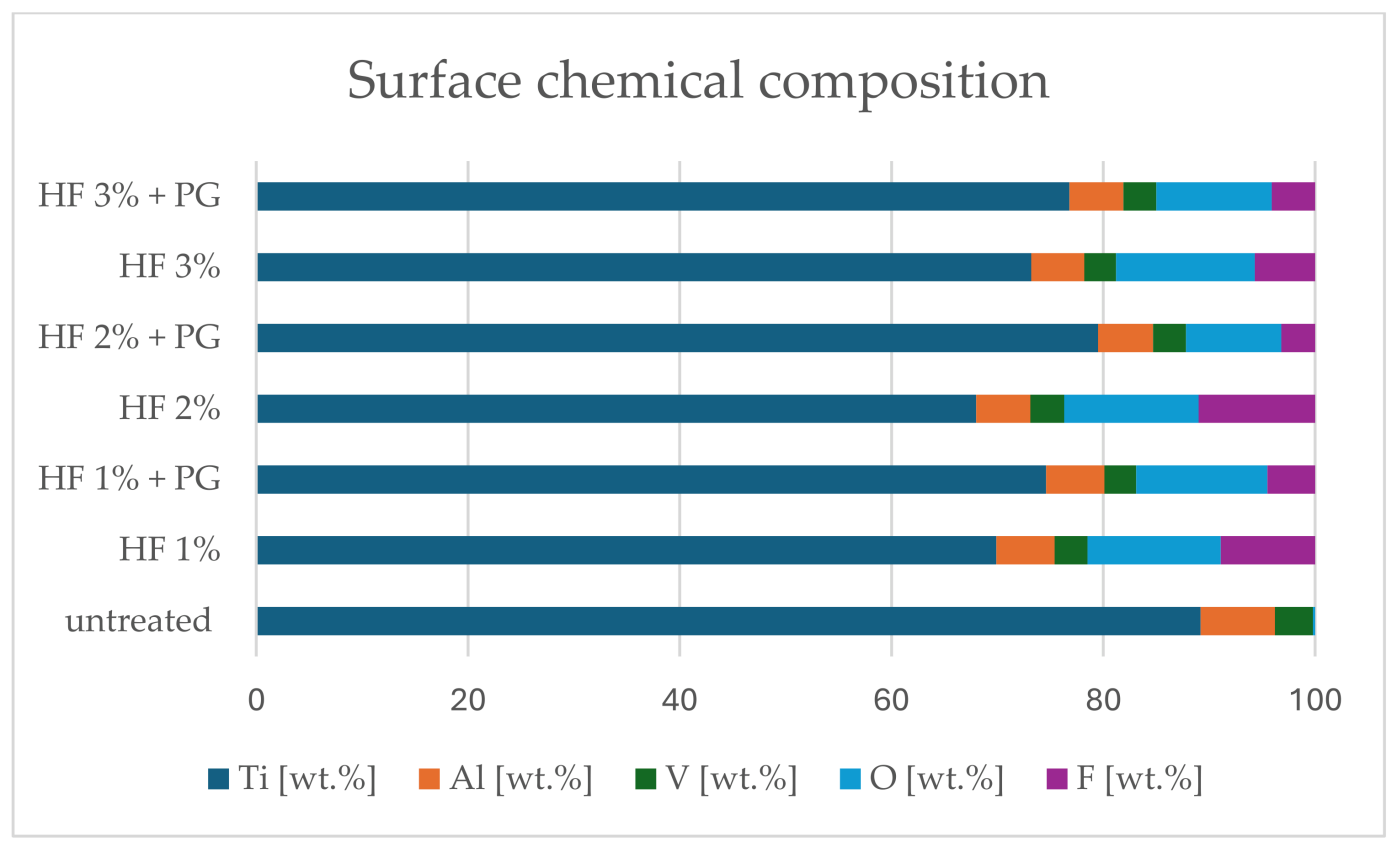
Surface chemical composition of printouts undergoing chemical etching.

The surface chemical composition of the 3D printed samples before and after chemical etching was summarized in Figure 3. The untreated sample exhibited a composition in line with common composition limits for Ti6Al4V alloy (Aluminum 5.50–6.75%; Vanadium: 3.50–4.50%; Titanium: balance). No fluorine was detected. The amount of oxygen was negligible that indicates only a thin native oxide layer on the surface of printouts. Chemical etching caused pronounced modifications in surface chemistry. For all HF treated samples, an increase in oxygen content and the incorporation of fluorine was visible.

Fluorine incorporation strongly depended on HF concentration, with the highest amount of that element recorded for samples etched in 2% HF (11.0 wt.%). Oxygen content reached values between 12.6 and 13.1 wt.% for solutions containing only water and HF, suggesting re-growth of the thick surface oxide layer after partial dissolution of titanium.

The addition of propylene glycol reduced fluorine incorporation compared to their glycol-free counterparts. The greatest reduction (about 70%) was achieved for solution containing 2% HF and the lowest (only 30%) for 3% HF solution. Simultaneously, oxygen content was reduced for glycol containing solutions that may reflect slowing surface oxidation in that type of etching mixture.

### 3.4. Wettability and Surface Free Energy

Chemical etching altered the wettability of solid 3D printouts. The untreated surface exhibited hydrophilic behavior, characterized by relatively low water contact angles (about 47°). After chemical treatment an increase in water contact angle was observed for all samples, indicating a transition toward more hydrophobic surface behavior. Samples etched in solutions without propylene glycol showed the highest water contact angles. Contact angle measurements with diiodomethane revealed the opposite trend, with etched surfaces showing decreased values compared to untreated samples, reflecting increased affinity toward non-polar liquids. The overall surface free energy was decreased as a result of etching, reaching the lowest values for solutions containing 3% HF (Table 5). Samples etched with propylene glycol exhibited slightly higher surface free energy values than their glycol-free counterparts across all investigated HF concentrations, although the contribution of the polar and dispersive components depended on the etching conditions.

### 3.5. Internal Structure

**Figure 4:**
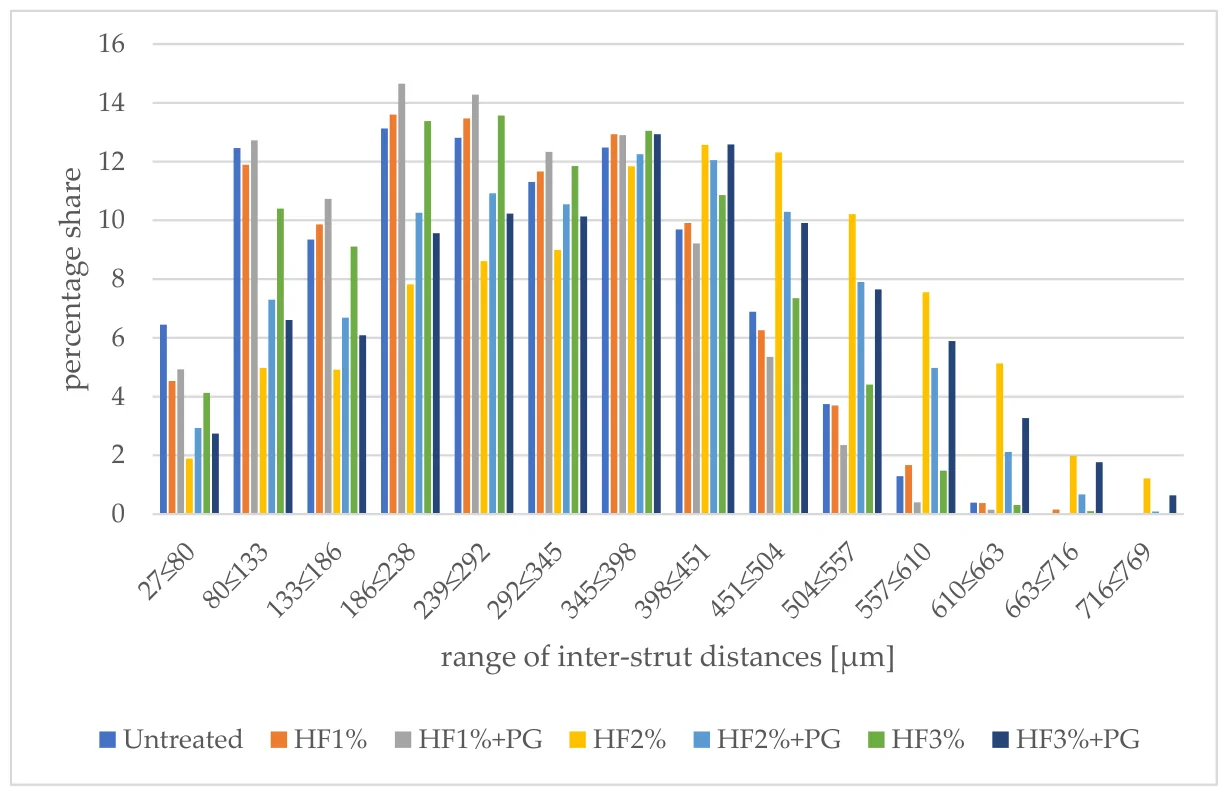
Distribution of inter-strut distances determined by micro-CT analysis for porous printouts in the as-built condition and after chemical etching in HF solutions with and without propylene glycol.

Micro CT analysis revealed that chemical etching affected the internal architecture of porous printouts. Changes in total porosity, strut thickness, and inter strut spacing were observed depending on the etching conditions (Figure 4 and Table 6). The untreated specimens were used as the internal reference for all structural comparisons, allowing the effects of chemical etching to be evaluated relative to the same manufacturing route rather than to the confidential nominal design parameters of the commercial implant architecture. Since one porous specimen was analyzed for each etching condition, the reported standard deviations represent the geometric heterogeneity within the analyzed specimen rather than experimental reproducibility among independent specimens.

The untreated material exhibited a total porosity of about 35%. It corresponded to mean wall thickness of 410 µm to and mean size of pores (distances between the walls) equal to 279 µm. The highest porosity of about 45% was achieved for samples HF 2% and HF 3% + PG. Etching affected also the size of pores. Untreated samples as well as HF 1% and HF 1% + PG showed almost 60% of pores for which the distance between walls was in the range of 300–600 µm and the pronounced fraction (about 35%) in the range of 155–265 µm. In the case of HF 2%, HF2% PG and HF 3% PG about 70% of pores was in the range 300–600 µm. The mean size of pores exceeding 350 µm was observed for HF 2%. However, as much as 19% distance between walls in range over 525 µm was observed only for HF 2% and HF 3% + PG. The next result in such range was equal to 15.7% for HF 2% + PG. The increase in pore size was concomitant with the reduction in strut thickness from a mean value of 410 µm for untreated samples to 390 µm for HF 2% and 394 µm for HF 3% + PG, respectively. The wall thickness parameter obtained from threshold-based Micro-CT segmentation represents the average diameter of the largest spheres that can be inscribed within the reconstructed solid phase. Consequently, localized strut thinning visible in SEM micrographs does not necessarily translate into a proportional decrease in the mean local-thickness value of the entire porous structure, as the Micro-CT analysis provides an averaged structural descriptor of the reconstructed specimen.

### 3.6. Biological Evaluation

**Figure 5:**
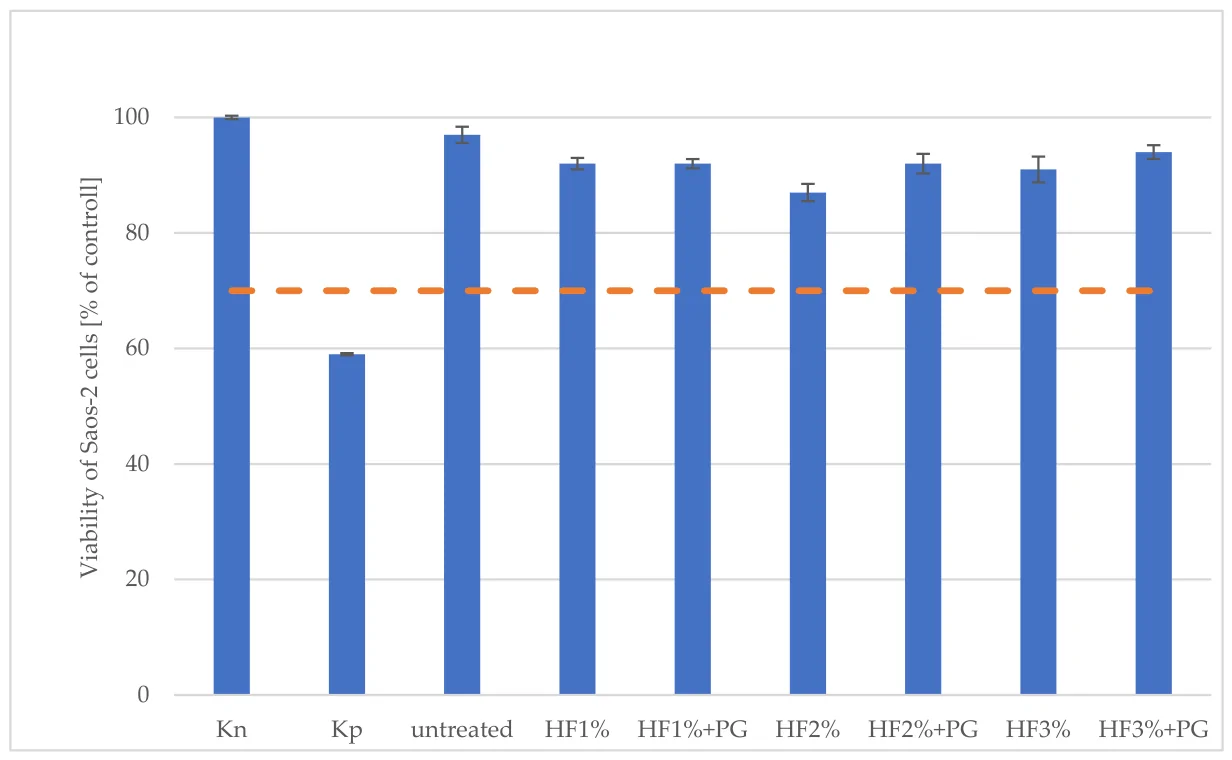
Cytotoxicity evaluation of untreated and chemically etched SLM fabricated printouts determined by XTT assay using *Saos-2* osteoblast like cells after 24 h exposure to sample extracts. Cell viability is expressed as a percentage relative to the negative control. The dashed line indicates the 70% viability threshold defined by ISO 10993 5; values above this limit indicate non-cytotoxic behavior.

The cytotoxic potential of the untreated and chemically etched printouts was evaluated using the XTT assay, and the results are presented in Figure 5. Cell viability values obtained for all tested samples exceeded the 70% threshold defined by ISO 10993 5, confirming the absence of cytotoxic effects under the applied testing conditions.

The highest cell viability was observed for the untreated sample, reaching approximately 97%. Chemical etching caused a slight reduction in cell viability, with the magnitude of the effect depending on the HF concentration and the presence of propylene glycol in the etching solution. In all examined cases results obtained for solutions containing glycol are not lower than in case of mixture of acid and water.

## 4. Discussion

The present study demonstrates that chemical etching using hydrofluoric acid is an effective post processing method for improving the surface quality of SLM fabricated Ti6Al4V structures; however, its effectiveness depend on the aggressiveness of the etching process. The results clearly indicate that the addition of propylene glycol allows better control over the etching process, mitigating excessive material loss while preserving favorable surface, structural, and biological properties required for biomedical implant applications. The concentration of propylene glycol was intentionally limited to 1 vol.% in order to evaluate whether even a minor modification of the etching medium could affect the etching behavior of HF solutions. The objective of the present study was not to optimize the glycol concentration but rather to verify the feasibility of this previously unexplored post-processing approach. Accordingly, no systematic optimization of propylene glycol concentration was performed, and identifying the optimal PG content remains a subject for future investigation. The reduction in material loss observed for all HF concentrations demonstrates that even a small addition of propylene glycol can substantially modify the etching kinetics of Ti6Al4V. For porous Ti6Al4V structures, the addition of propylene glycol reduced the mass loss by approximately 40–50% for both 2% and 3% HF solutions. Among all examined samples the mass loss did not exceed 31%, while the literature indicates even a 62% loss of mass in printed samples treated with just 1.3% HF and the addition of 9% HNO_3_, but with a longer etching time [21]. It should nevertheless be acknowledged that the reported mass-loss values reflect the complete post-processing procedure, including the additional cleaning step (in isopropyl alcohol) performed before etching, and should therefore be interpreted within this methodological context. An alternative solution to the problem of weakly bonded powders may be the use of a different method, such as a plasma-based technique, which causes only about a 1% mass loss [22].

EDS analysis revealed that HF etching substantially modifies the surface chemical composition of Ti6Al4V, introducing fluorine and increasing oxygen content. These changes are associated with partial dissolution of the native titanium oxide layer followed by re-oxidation and probable formation of fluoride-containing surface species.

The degree of fluorine incorporation varied with HF concentration in the range of 3.2 to 11%. Importantly, the addition of propylene glycol reduced fluorine content at all HF concentrations. This observation suggests that propylene glycol may moderate the etching process. A possible explanation is that the addition of propylene glycol increases the viscosity of the solution and alters the local mass transport of reactive species, thereby influencing the dissolution kinetics. However, this mechanism remains hypothetical, as viscosity, mass transport, and dissolution kinetics were not directly measured in the present study. At the same time, aluminum and vanadium contents remained relatively stable, indicating that etching primarily affected the surface oxide layer rather than inducing preferential dissolution of alloying elements.

While higher HF concentrations effectively substantially reduce the amount of weakly bonded powder particles typical of the SLM process, aggressive etching without propylene glycol led to surface thinning and localized material loss. In contrast, HF solutions containing propylene glycol provided effective powder removal with reduced formation of surface defects. Reduction of surface roughness after chemical etching was observed for all treated solid samples. In glycol-free solutions, increasing HF concentration caused a gradual smoothing of the surface, as evidenced by decreasing Ra and Rz values. This effect is consistent with preferential dissolution of sharp asperities formed during layer-wise melting in SLM.

Wettability measurements revealed a transition from hydrophilic behavior of the untreated surface toward more hydrophobic characteristics after etching. There is no universal trend of change of contact angle due to chemical etching of titanium. Kongsong et al. reported increase in hydrophobicity corelated with time of etching [23] and values of water contact angle on similar level (of about 110°) for coincident etching time. Other studies conducted with different concentrations of acid or time also show similar tendency [24]. On the other hand, some authors describe also reduction of water contact angle due to acid post-processing [25,26]. Shift towards more hydrophobic features is rather unfavorable from the perspective of bone forming cells response [27]. The hydrophobic surface may favor the adsorption of serum proteins. However, the biological response depends not only on the amount of adsorbed proteins but also on their conformation and bioactivity. The potentially less favorable, for bone forming cells, wettability may be balanced by more favorable pore size. It should be noted that roughness, wettability, and surface free energy were measured on solid SLM specimens, which served as a comparative surface model because these measurements cannot be reliably performed using standardized methods on the complex geometry of porous lattice structures. Therefore, these results should be interpreted as comparative indicators of the influence of the etching conditions rather than as direct quantitative characteristics of the porous struts, although they provide useful insight into the relative surface modifications induced by the different post-processing treatments. An additional limitation of the present study is that no surface-sensitive chemical analysis, such as XPS, was performed after the final washing procedure to verify the possible presence of residual propylene glycol-derived organic species. Such analysis would allow a clearer distinction between wettability changes resulting from modified etching chemistry and those potentially associated with residual surface contaminants.

Micro CT analysis demonstrated that chemical etching influences not only surface features but also the internal architecture of porous Ti6Al4V structures. These structural observations should be interpreted as changes identified within representative specimens for each etching condition rather than as statistical variability between independent specimens. Moderate increases in total porosity and size of pores were observed predominantly for samples 2% HF and HF3% + PG. These results are highly relevant from a biological perspective. Fully interconnected open porosities in the range of 50–70% combined with mean pore sizes of 300–600 µm are widely considered favorable for bone tissue ingrowth, vascularization, and long term osseointegration [28,29]. Mukasheva, et al. [30] claims also that porosities of 200–400 µm, may enhance nutrient diffusion and angiogenesis in scaffolds for bone regeneration. On the other hand C Torres-Sanchez et al. [31] indicated that The pore size in range of 212–300 μm and about 45% porosity is the least supportive to cell proliferation in multiple day time intervals. The micro CT results indicate that, for selected etching conditions—especially HF solutions modified with propylene glycol—the porous structures approach towards the range favorable for bone ingrowth. Importantly, etching with propylene glycol preserved a more uniform distribution of wall thickness and inter strut distances, which may help preserve the structural characteristics of the porous architecture, although the mechanical implications were not directly evaluated in the present study. Micro-CT results should therefore be interpreted as averaged structural descriptors of the analyzed specimens rather than direct measurements of localized strut thinning observed in SEM images, since the local-thickness parameter is derived from threshold-based segmentation of the reconstructed solid phase. Excessive etching of struts may lead to decrease of printout mechanical properties or its overall constructional integrity as stated in the literature [32,33].

XTT cytotoxicity assays demonstrated that all investigated samples were non-cytotoxic toward *Saos-2* osteoblast like cells, with cell viability exceeding the 70% threshold defined by ISO 10993 5. Although a slight reduction in cell viability was observed after chemical etching—particularly for samples etched in solutions of only water and acid —these changes were minor and did not indicate cytotoxic effects under the conditions of the extract-based XTT assay. This is in line with numerous scientific data showing that fluorine present on the surface of titanium implant surface does not adversely affect [14,34] or even can improve bone regeneration by positive stimulation of osteogenic cells [35,36]. The cytotoxicity observed in the extract-based XTT assay may be influenced by multiple factors, including fluoride incorporation, wettability, and surface topography; however, the present assay does not allow the individual contribution of these factors to be distinguished. The improved cell viability observed for samples etched with propylene glycol may be associated with differences in fluorine content, surface free energy, or surface roughness; however, the extract-based XTT assay does not allow the individual contribution of these factors to be distinguished. Previous studies indicate that implant wettability affects protein adsorption, while surface roughness may influence subsequent biological interactions [37,38]. These aspects were not directly evaluated in the present study and therefore require dedicated cell-on-surface experiments.

Taken together, the results demonstrate that chemical etching is a powerful but highly sensitive post processing technique for SLM fabricated Ti6Al4V. While high HF concentrations resulted in substantial reduction of loosely bound powder particles, they also introduce risks of excessive material loss, surface damage, and mechanical degradation.

The incorporation of propylene glycol into HF etching solutions emerges as an effective strategy for controlling etching kinetics. This approach enables efficient powder removal while reducing material loss and producing structural characteristics that, according to the literature, fall within ranges considered favorable for bone ingrowth. Furthermore, all investigated samples remained non-cytotoxic in the extract-based XTT assay. The conditions considered potentially supportive for bone cell ingrowth characteristic for HF solutions are in line with majority of scientific claims [13,16]. The addition of glycols appears to be an interesting aspect warrants further investigation, particularly from the perspective of reducing the threat of samples over-etching.

Beyond titanium alloys, biodegradable magnesium- and zinc-based scaffolds have attracted increasing attention for bone regeneration owing to their bioactive behavior and gradual degradation in vivo. In these materials, HF treatment is primarily used as a fluoride conversion treatment that promotes the formation of MgF_2_-based protective layers regulating the degradation process, often under substantially different processing conditions involving higher HF concentrations and longer treatment times than those applied in the present study [39,40,41]. In contrast, the HF-based treatment investigated here was intended to remove loosely bound powder particles and control the surface and porous architecture of SLM-fabricated Ti6Al4V rather than to produce a protective conversion coating. Nevertheless, the general concept of tailoring surface modification through the composition of the processing medium may provide useful inspiration for future studies on other metallic scaffold materials, provided that the processing chemistry is adapted to the specific characteristics of each alloy.

## 5. Conclusions

The present exploratory study investigated the effect of hydrofluoric acid solutions modified with propylene glycol on the post-processing of SLM-fabricated Ti6Al4V structures intended for orthopedic applications. The results demonstrated that hydrofluoric acid effectively removed partially fused and weakly bonded powder particles characteristic of the SLM process. However, increasing HF concentration increased material loss, particularly in porous structures. The addition of 1 vol.% propylene glycol substantially reduced the aggressiveness of the etching process, decreasing mass loss by up to 50% while maintaining effective powder removal.

Chemical etching altered surface morphology, roughness, wettability and chemical composition of modified samples. The incorporation of propylene glycol reduced fluorine accumulation on the surface compared with glycol-free HF solutions, while preserving the smoothing effect achieved due to etching. Micro-CT analysis revealed that selected etching conditions modified the internal architecture of porous structures. Altering the range of pore sizes as well as porosity itself contributed to conditions considered more favorable for bone tissue ingrowth and vascularization. At the same time, glycol-containing solutions limited excessive degradation of struts. All investigated samples fulfilled the ISO 10993-5 criterion for non-cytotoxic materials. The preliminary biological evaluation confirmed that the proposed post-processing approach did not induce cytotoxic effects under the conditions of the extract-based XTT assay. Overall, this exploratory study demonstrates the feasibility of incorporating propylene glycol into HF-based etching solutions as a strategy for controlling the post-processing of additively manufactured Ti6Al4V. The proposed approach enables efficient powder removal while reducing material degradation and preserving desirable surface characteristics, the observed structural features, and the non-cytotoxic response demonstrated in the XTT assay. The results indicate that HF–propylene glycol systems represent a promising direction for further optimization of post-processing protocols for porous 3D printed titanium implants.

## Figures and Tables

**Table 1:** Printing parameters used for samples preparation.

Printing Parameter	Value
Mean layer thickness	45 μm
Laser power	500 W
Printout accuracy	±0.2%

**Table 2:** Mass loss of porous SLM fabricated Ti6Al4V samples after chemical etching in HF solutions with and without propylene glycol. Values represent mean ± standard deviation (three consecutive weighings of the same specimen).

Sample	Mass Before Etching [g]	Mass After Etching [g]	Mass Loss [%]	Standard Deviation [%]
HF 1%	1.96	1.87	4.36	0.82
HF 1% + PG	1.97	1.91	3.33	0.07
HF 2%	1.95	1.62	17.21	0.78
HF 2% + PG	1.96	1.77	9.85	0.13
HF 3%	1.98	1.37	30.91	1.04
HF 3% + PG	1.96	1.66	15.62	0.51

**Table 3:** Mass loss of solid SLM fabricated Ti6Al4V samples after chemical etching in HF solutions with and without propylene glycol. Values represent mean ± standard deviation (three consecutive weighings of the same specimen).

Sample	Mass Before Etching [g]	Mass After Etching [g]	Mass Loss [%]	Standard Deviation [%]
SHF 1%	3.46	3.43	0.79	0.07
SHF 1% + PG	3.47	3.45	0.75	0.08
SHF 2%	3.48	3.43	1.51	0.16
SHF 2% + PG	3.50	3.45	1.45	0.15
SHF 3%	3.49	3.41	2.47	0.14
SHF 3% + PG	3.49	3.41	2.18	0.13

**Table 4:** Surface roughness parameters of solid SLM fabricated Ti6Al4V samples after chemical etching in HF solutions of different concentrations, with and without the addition of propylene glycol (PG). Values represent mean ± standard deviation based on ten measurements.

Sample	Ra [µm]	SD [µm]	Rz [µm]	SD [µm]
Untreated	5.39	0.85	30.96	4.55
SHF 1%	5.01	0.35	27.76	2.32
SHF 1% + PG	4.48	0.61	23.56	2.83
SHF 2%	4.84	0.34	26.23	3.41
SHF 2% + PG	4.63	1.12	25.19	3.06
SHF 3%	4.61	0.53	24.15	3.69
SHF 3% + PG	4.78	0.90	25.56	3.99

**Table 5:** Contact angles (water and diiodomethane) and surface free energy of solid SLM printouts after HF-based chemical etching with and without propylene glycol.

Sample	Water Contact Angle [°]	Standard Deviation	Dijodomethane Contact Angle [°]	Standard Deviation	Surface Energy [mJ/m^2^]	Polar Component [mJ/m^2^]	Dispersive Component [mJ/m^2^]
untreated	47.42	3.26	62.65	2.96	38.4	11.4	27.0
SHF 1%	114.32	10.05	47.9	2.89	37.8	2.4	35.4
SHF 1% + PG	109.88	11.61	44.84	2.77	38.1	1.0	37.1
SHF 2%	95.92	9.12	49.98	4.63	34.3	0.0	34.3
SHF 2% + PG	91.66	6.16	46.58	4.79	36.4	0.2	36.2
SHF 3%	113.10	10.79	56.44	5.26	33.9	3.3	30.6
SHF 3% + PG	102.86	3.51	53.98	2.80	34.2	0.5	33.7

**Table 6:** Results of microtomography analysis of tested 3D printouts.

Sample	Mean Wall Thickness [µm]	Standard Deviation	Mean Distance Between Walls [µm]	Standard Deviation	Total Porosity [%]
Untreated	410	145	279	138	35.1
HF 1%	474	144	285	135	33.8
HF 1% + PG	483	147	269	126	33.3
HF 2%	390	111	392	159	46.9
HF 2% + PG	416	125	343	150	40.0
HF 3%	477	138	294	135	35.8
HF 3% + PG	394	123	256	158	43.8

## Data Availability

The original contributions presented in this study are included in the article. Further inquiries can be directed to the corresponding author.

## Abbreviations

The following abbreviations are used in this manuscript:

AM	Additive Manufacturing
SLM	Selective Laser Melting
EBM	Electron Beam Melting
HF	Hydrofluoric Acid
PG	Propylene Glycol
SEM	Scanning Electron Microscopy
EDS	Energy-Dispersive X-ray Spectroscopy
X-ray micro-CT	X-ray Micro-Computed Tomography
SFE	Surface Free Energy
XTT	2,3-bis(2-Methoxy-4-nitro-5-sulfophenyl)-2H-tetrazolium-5-carboxanilide Assay
ISO	International Organization for Standardization
ELI	Extra Low Interstitial
HCl	Hydrochloric Acid
HNO_3_	Nitric Acid
TiO_2_	Titanium Dioxide
rpm	Revolutions per Minute
Saos-2	Human Osteoblast-Like Osteosarcoma Cell Line
HUVEC	Human Umbilical Vein Endothelial Cells
